# Polysaccharide Fabrication Platforms and Biocompatibility Assessment as Candidate Wound Dressing Materials

**DOI:** 10.3390/bioengineering4010001

**Published:** 2017-01-18

**Authors:** Donald C. Aduba, Hu Yang

**Affiliations:** 1 Department of Biomedical Engineering, Virginia Commonwealth University, Richmond, VA 23284, USA; adubad@vcu.edu; 2 Department of Chemical and Life Science Engineering, Virginia Commonwealth University, Richmond, VA 23284, USA; 3 Department of Pharmaceutics, Virginia Commonwealth University, Richmond, VA 23298, USA; 4 Massey Cancer Center, Virginia Commonwealth University, Richmond, VA 23298, USA

**Keywords:** wound healing, wound dressing, foam, nanofiber, hydrogel, wound management, skin, polysaccharide

## Abstract

Wound dressings are critical for wound care because they provide a physical barrier between the injury site and outside environment, preventing further damage or infection. Wound dressings also manage and even encourage the wound healing process for proper recovery. Polysaccharide biopolymers are slowly becoming popular as modern wound dressings materials because they are naturally derived, highly abundant, inexpensive, absorbent, non-toxic and non-immunogenic. Polysaccharide biopolymers have also been processed into biomimetic platforms that offer a bioactive component in wound dressings that aid the healing process. This review primarily focuses on the fabrication and biocompatibility assessment of polysaccharide materials. Specifically, fabrication platforms such as electrospun fibers and hydrogels, their fabrication considerations and popular polysaccharides such as chitosan, alginate, and hyaluronic acid among emerging options such as arabinoxylan are discussed. A survey of biocompatibility and bioactive molecule release studies, leveraging polysaccharide’s naturally derived properties, is highlighted in the text, while challenges and future directions for wound dressing development using emerging fabrication techniques such as 3D bioprinting are outlined in the conclusion. This paper aims to encourage further investigation and open up new, disruptive avenues for polysaccharides in wound dressing material development.

## 1. Introduction

Countless numbers of wounds are generated worldwide each year due to surgical procedures as well as trauma and as the result of non-healing ulcers and burns. Wounds demand time for care and treatment from a substantial number of medical staff in already heavily burdened hospitals. The annual wound care products market was estimated to reach $15.3 billion in 2010, illustrating its global clinical demand [1]. Wound dressings have become increasingly critical in promoting wound healing and wound management. Many types of materials have been utilized to develop wound dressings and have been commercialized in the market summarized in [2]. Wound dressings provide an environment for the wound to heal at the maximum rate under particular pathological conditions while achieving a cosmetically acceptable appearance [3]. They are designed to protect the wound from the external environment while keeping it moist for proper healing. Modern wound dressings are designed to absorb wound exudate to manage the wound healing process. There are greater demands for the wound dressing to actively play a role in the wound healing process. Therefore, incorporation of bioactive components in wound dressings helps improve wound exudate absorption and remove the etiologies of exudate production [4]. 

Bio-derived polymers such as polysaccharides have been widely used in wound dressing development because of their natural abundance in plants and production in the body. For example, chitosan is widely present in shrimp and crab shells while arabinoxylan polysaccharides are present in wheat food products. Polysaccharides are also biocompatible, non-immunogenic, and anti-microbial [5,6,7]. Therefore, polysaccharide dressings may encourage more efficient wound healing. Polysaccharides are structurally diverse in terms of molecular weight, charge, and chemical composition, and they provide a wide range of structural parameters and properties for manufacturing wound dressings specific to the wound etiology. In this paper, the authors present an overview of literature reporting polysaccharides that have been fabricated and characterized to assess their applicability as wound dressing materials. The second section will discuss the wound healing process to provide the foundation and rationale for selecting polysaccharides as wound dressing materials. In the third section, a summary of polysaccharide fabrication processing techniques using hydrogel crosslinking and electrospinning fiber scaffolds will be discussed. The fourth section provides a literature survey of polysaccharide platforms to assess biocompatibility at a preclinical level. The conclusion of this review highlights the utility of polysaccharides, discussing challenges that need to be overcome and new fabrication avenues for these natural polymers to become a candidate wound dressing material. 

## 2. The Wound Healing Process

**Figure 1:**
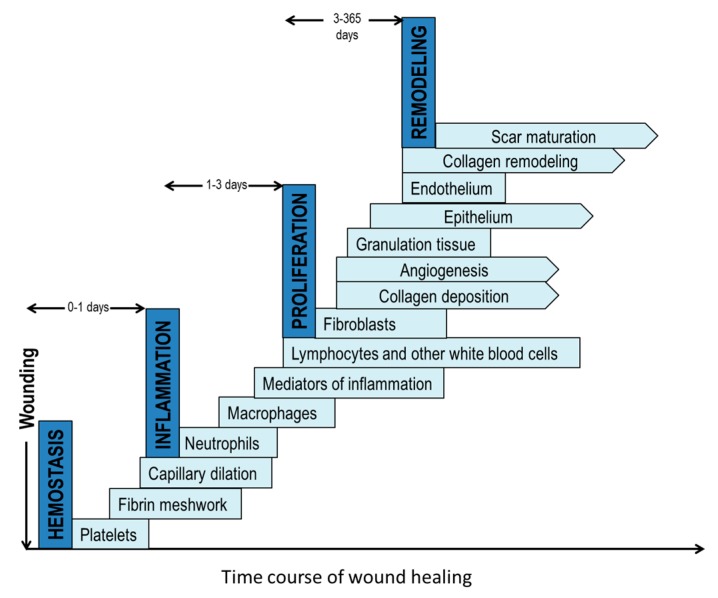
The four phases of normal wound healing: (**1**) homeostasis; (**2**) inflammation; (**3**) proliferation and (**4**) remodeling. Each step has many components. The pointed edge depicts an ongoing process.

Wound healing is a highly complex process as it requires a sequence of biochemical and cellular events involving extracellular matrix components, cells, and extracellular molecules [8,9]. The proper synergy between these events and cellular components will determine if the healing process is successful or becomes delayed in a chronic state (Figure 1) [10,11]. There are four stages in a normal wound healing process: hemostasis, inflammation, proliferation and remodeling [2,12]. Hemostasis begins immediately following an injury. Platelets are then recruited to the injury site and play an important role in forming a clot to minimize the bleeding. The clot provides a temporary matrix to recruit and home the cells involved in the subsequent wound healing response [2,13]. The inflammation phase begins about one day after the injury. During this phase, neutrophils are recruited to perform phagocytosis to destroy and remove bacteria, pathogens, and cell debris. In the inflammatory phase, approximately two to three days after injury, tissue macrophages derived from blood monocytes continue the phagocytic activity. They also play a role in attracting and activating fibroblasts, keratinocytes, and endothelial cells [8,14]. The subsequent proliferation stage, typically starting on the third-day post-injury, is characterized with the appearance of fibroblasts at the wound and the production of new extracellular matrix, made mostly of collagen to rebuild the tissue. Concurrently, new blood vessels and granulation tissue form as a result [8]. In the final remodeling phase, new epithelium forms along with the transition of granulation tissue to a mature scar, which is less cellular and vascular but has a significantly high tensile strength. The remodeling stage may last a year. 

Depending on the healing time frame, wounds can be classified as acute or chronic. Acute wounds are able to heal by timely progression through the normal stages of healing. However, there exist multifactorial and complex pathophysiological circumstances that lead to chronic wounds, which may undergo a prolonged healing process in one or more stages or fail to heal. For instance, chronic non-healing wounds such as pressure ulcers have an abnormal and prolonged inflammatory phase during which a large number of highly activated neutrophils release an excessive quantity of degradative enzymes that destroy the tissue that is being repaired. In order to ensure an efficient wound healing process, the wound dressing platform and material selection are critical. 

Polysaccharides are an intriguing class of materials for wound healing optimization because of their natural origin, which has led to significant research into their processing for functional wound dressing materials. Natural polysaccharides play a role in wound healing because of their ability to promote non-specific activation of the immune system by activating macrophages that clean up the wound site after injury. In many polysaccharides, a beta 1,3 d-glucan linker is present for macrophage receptors binding to initiate would healing [15]. Natural polysaccharides contain glycosaminoglycans that are present in the extracellular matrix. They function during wound healing by binding to proteins at hierarchical specificity and are involved mainly in the development, cell differentiation, cell adhesion, cell signaling and cell-matrix interactions [16]. Glycosaminoglycans have been demonstrated to improve the wound healing process through re-epithelialization and increased vascularization [17]. The next section will discuss polysaccharides utilized as hydrogels and electrospun fibers, the two primary platforms used for fabricating wound dressing materials.

## 3. Fabrication Platforms of Polysaccharides as Wound Dressing Materials

### 3.1. Hydrogels

Hydrogels are crosslinked polymeric dressings and insoluble in aqueous media. They are excellent platforms for wound dressing applications because they swell significantly to help with wound exudate absorption. Also, their hydrophilic properties enable the hydrogel to keep the wound bed moist. They provide a cool and non-adherent surface due to the hydrating properties of the gel, preventing heat absorption and encouraging wound debridement and comfort for the patient. Hydrogels can deliver antimicrobials for sustained action against infected wounds. Hydrogels are very flexible and conform to a variety of conditions [18,19,20,21]. Polymer properties such as molecular weight, charge, and crosslinking density all play a role in modulating the degree of hydrogel swelling in aqueous solutions. Typically, hydrogels with high molecular weights and crosslinking densities are stiff and rigid with high modulus values [22,23]. Meanwhile, solute diffusion out of hydrogels is controlled by their crosslinking density and mesh sizes [24,25]. These properties in polysaccharide gels have been modeled by Berger et al. who established the relationship between covalent and ionic bonding with the degree of crosslinking in chitosan [26]. Polysaccharides in hydrogel formulations have been used in many applications because they are highly versatile, complex polymers that are readily available and can be easily manipulated into gels. They are natural, non-toxic while exhibiting immunomodulatory properties [27]. Polysaccharide hydrogels have been predominantly used as drug delivery carriers but are gaining attraction as wound dressing materials because of the similar structure it has to the extracellular matrix. A variety of polysaccharides have been used for tissue engineering and drug delivery such as alginate, gellan, dextran, hyaluronic acid, pullulan, etc. [28]. The charge in the polymer system (basic, neutral or acidic), nature of crosslinking, and type of polysaccharides need to be considered during the fabrication step to form effective hydrogel wound dressings [29]. The remaining portion of the section will discuss principles of covalent and ionic crosslinking mechanisms used in polysaccharide hydrogel formation.

#### 3.1.1. Covalent Crosslinking

Covalent crosslinking is an irreversible process in which permanent bonds are made within the hydrogel structure. Covalent crosslinking of chitosan hydrogels is straightforward using either small molecules, light, enzyme catalysts or monomers to create more stable networks. This crosslinking method may be toxic because of the by-products created from the reaction [30]. Therefore, removal of these by-products is necessary prior to wound dressing application. One strength of covalent crosslinking is that it enables water and drugs to be absorbed without compromising the mechanical integrity of the hydrogel [26]. This is ideal as a wound dressing because its strength allows the incorporation of agents such as antimicrobials and growth that later diffuse to the wound site for improved healing. Hyaluronic acid is a linear polysaccharide that is made up of N-acetyl-d-glucosamine and glucuronic acid. Hyaluronic acid has recently gained more attention for wound dressing formation because of its natural presence in the extracellular matrix and involvement in the inflammation and proliferation stages of wound healing [31]. Hyaluronic acid currently has clinical uses as wound dressings [31], skin substitutes [32], and joint lubricants [33]. Hyaluronan-based biomaterial (HYAFF-11), a commercialized biocompatible hyaluronic acid matrix, has improved mechanical integrity upon swelling by implementing an esterification crosslinking process [31]. This enables swelling up to 1000 times its weight for highly exudative wounds while retaining its integrity upon application. Other examples in the literature have used hyaluronic acid hydrogels upon crosslinking with the use of glutaraldehyde and 1-ethyl-3-(3-dimethylaminopropyl) carbodiimide (EDC) reagents [34]. One group used hyaluronic acid functionalized with adipic dihydrazide and crosslinked with poly-ethylene glycol (PEG) propionaldehyde to create a film. It was successfully applied to deliver anti-microbial and anti-inflammatory agents in vitro, showing promise as a wound dressing [35]. Leach et al. have employed photopolymerization to crosslink hyaluronic acid with glycidyl methacrylate groups to from biocompatible hydrogels [36]. Eng et al. had investigated effects of thiol-functionalization of hyaluronan at 20% and 44% substitution density to modulate PEG-diacrylate gel stiffness. The number of crosslinking chemistries that form hyaluronic acid gels are not all covered in this review and can be found in a hyaluronic progress review written by Burdick et al. [37]. 

**Figure 2:**
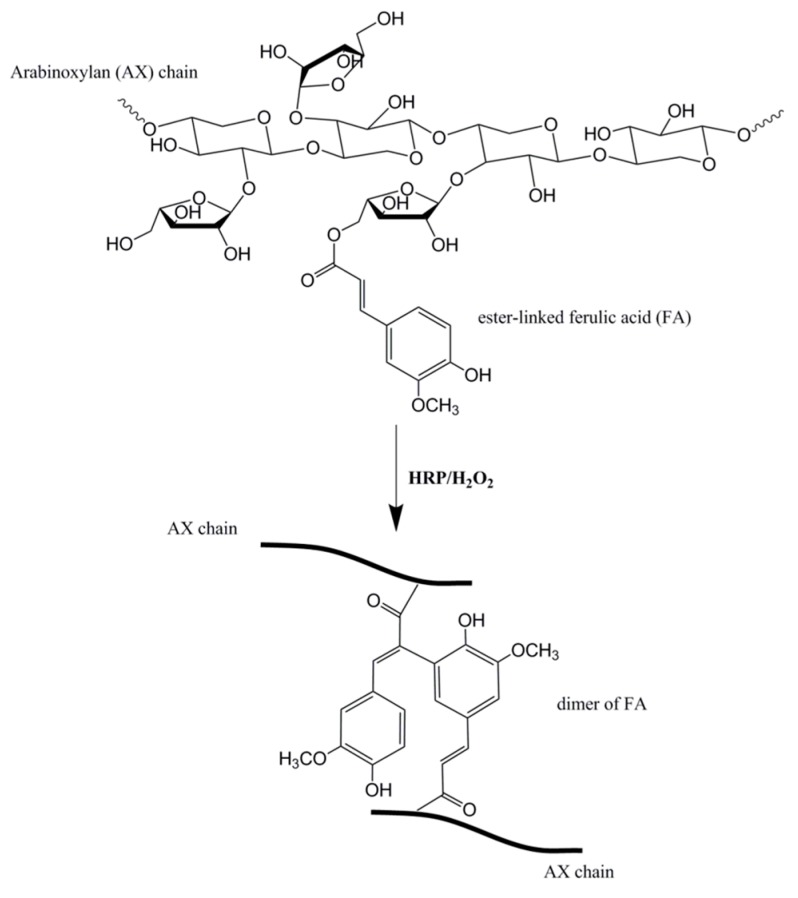
Arabinoxylan ferulate (AXF) structure composed of three components: xylose backbone substituted to arabinose sugar units, one of which is estyyanger-linked to ferulic acid. It can be crosslinked using HRP/H_2_O_2_. Figure adapted from reference [44] with permission.

While hyaluronic acid is a popular polysaccharide for wound dressings, other polysaccharides have been investigated as hydrogel wound dressing platforms. A systematic study of highly absorbent pullulan polysaccharide hydrogels evaluated crosslinking density of cystamine to modulate tensile strength, swelling and mass loss [38]. Pullulan is a polysaccharide produced by different strains of *Aureobasidium* bacteria. It is a linear mixed linkage α-d-glucan consisting mainly of maltotriose units interconnected via α-(1→6) linkages [39]. The study indicated that covalent crosslinking of an organic disulfide agent, cystamine can tune the physical properties such as tensile strength and swelling ratio of the pullulan hydrogel for the desired release of antibacterial agents. A recent study by Jiang et al. indicated that hypromellose succinate-chitosan hydrogels prepared using EDC/NHS (N-hydroxysuccinimide) crosslinker exhibited good cytocompatibility after extracting catalyst by-products by dialysis [40]. Arabinoxylan (AX) is a neutral non-starch polysaccharide derived from cereal grains such as wheat with anti-oxidant properties that can be crosslinked by chemical means using enzymatic precursors [41,42]. They are water extractable and are comprised of a xylose backbone substituted onto arabinose units. Arabinoxylan ferulate (AXF) is arabinoxylan with ferulic acid substituted onto its arabinose monomer that can be readily crosslinked into gels via enzymatic reaction, for instance using horseradish peroxidase (HRP) and hydrogen peroxide (H_2_O_2_) (Figure 2). They function by creating an ester bond between ferulic acid and arabinose units to form a dimer which crosslinks the arabinoxylan chains together [43]. While arabinoxylan is naturally derived and relatively abundant, they have yet to be fully explored as a wound dressing material. Arabinoxylan is a good candidate polysaccharide hydrogel wound dressing because it is very hydrophilic and highly absorbent. The flexibility of arabinoxylan chains enables fluid and solute movement in and out of the delivery system based on the gel’s degree of crosslinking [41]. Experiments revealed arabinoxylan hydrogels to have a two-and-a-half-fold increase in swelling when introduced into water [43]. Lyophilized arabinoxylan gels fabricated in our study have demonstrated swelling ratios above 20 after 48 h [44]. Arabinoxylan is an intriguing polysaccharide for hydrogel wound dressing formation because it does not require the use of toxic organic solvents but instead uses water instead for solubilizing before crosslinking. 

#### 3.1.2. Ionic Crosslinking

**Figure 3:**
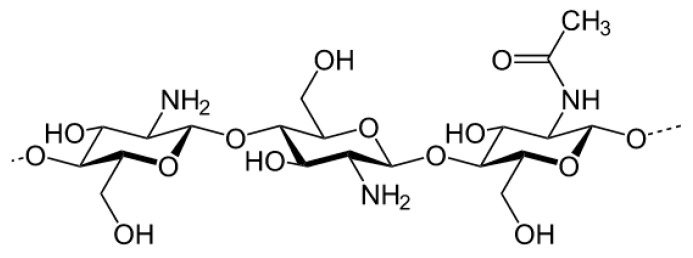
Chitosan structure.

Ionic crosslinking in hydrogels is a reversible process that permits greater swelling and pH-dependent swelling compared to covalently crosslinked hydrogels [26]. Ionic crosslinking uses ionic molecules to create a bridge within the polymer network. In the case of polysaccharides such as chitosan, their positive ions are crosslinked in the network by negatively charged crosslinkers such as metallic anions [45,46] or phosphate bearing groups [47]. These capabilities can be realized for application as wound dressing materials whose absorption and rate of diffusion can be tailored by acidity at the wound site. Chitosan is a prominent example of a polysaccharide that can be crosslinked ionically as it is a derivatized version of chitin, an exoskeleton component of crustacean shells. Chitosan constitutes a β 1-4 linked d-glucosamine and *N*-acetyl-d-glucosamine units (Figure 3). Chitosan hydrogels can be physically mixed into stable networks by introducing anionic ions or macromolecules to neutralize the positively charged chitosan and induce electrostatic attraction within the gelatinized network. Secondary bonding, hydrophobic-hydrophilic interactions, and thermo-responsive gelation can also take place in chitosan hydrogels depending on what monomers or catalysts are added to it [30]. Ionic crosslinking is a relatively safe technique to use for fabricating biocompatible hydrogels without toxic catalysts. While this method is non-toxic, there is the lack of long-term stability after physical crosslinking and should only be used for short-term applications. 

Other polysaccharides that undergo ionic crosslinking such as alginate is anionically charged and readily forms a hydrogel upon addition of divalent cations such as Ca^2+^. Alginate is made up of mannuronic acid (M) and guluronic acid (G) units derived from brown seaweed [16]. Alginate dressings have excellent absorption properties and can be used to treat wounds that create a high volume of exudate. They are able to absorb 15 to 20 times their weight and their gelling capability and mechanical strength of alginate dressings can be modulated by varying the M/G ratio [48]. Alginate hydrogels with high M-block content have high fluid absorption capacity but low mechanical strength. Conversely, alginate hydrogels with high G-block content have high strength but low water absorption capacity [49]. Alginates are generally suitable for all stages of wound healing. However, they are not suitable for dry wounds with little exudate [2,48]. The utility of alginate as a wound dressing is further substantiated with its role in activating macrophages to accelerate chronic wound healing [50]. Gellan gum is a water-soluble anionic polysaccharide produced by *Sphingomonas elodea* bacterium. Gellan’s chemical structure is based on a tetrasaccharide repeat unit of (1-3)-β-d-glucose, (1-4)-β-d-glucuronic acid, (1-4)-β-d-glucose, and (1-4)-α-l-rhamnose as the backbone. Similar to alginate, gellan can form gels when mixed with divalent ions. Its mechanical strength is dependent on the degree of acylated groups [28]. A higher degree of acylation tends to generate softer and more elastic gels. Also, gellan has thermoresponsive capabilities as gels whose molecular structure becomes ordered and disordered upon cooling and heating, respectively [51]. Although gellan has primarily been explored as drug delivery carriers, use of gellan as a wound dressing has been reported recently [52,53,54,55].

### 3.2. Electrospinning

Electrospinning is another major method to fabricate polysaccharide wound dressing materials. It was first patented by Anton Formhals in 1934 as a technique to create non-woven fibers using a voltage gradient between a fine syringe nozzle and collecting mandrel [56]. Specifically, the polymer solution ejected from the nozzle has an applied charge induced by a high voltage power supply. The applied charge in the solution overcomes its surface tension to create a jet that propels across space and deposit dry fibers at the collecting mandrel to create a non-woven fiber sheet. Electrospun nanofibers are an attractive platform as a wound dressing material because of their high surface to volume ratio and porosity that allows moisture and exudate transport between the dressing and injury site [57]. The high porosity of nanofiber dressings allows greater absorption of wound exudate than film dressing formulations [58]. In addition, the nanofiber’s high porosity provides an environment where cells can exchange oxygen and inhibit bacterial permeation at the wound-nanofiber interface [57]. Nanofiber wound dressings are highly flexible and conform to the shape of the wound because of their very fine fiber diameter. This provides better patient compliance and comfort [57]. Beyond the physical characteristics, nanofibers express or maintain biological functionality after integrating bioactive components such as therapeutics, growth factors, and antifungals to enhance the wound healing process [57]. These bioactive agents can be homogeneously distributed within the nanofiber scaffold and their nano-scale morphology encourages cell attachment and proliferation for extracellular matrix production [5,59].

To successfully fabricate electrospun fibers, the properties of polysaccharide, solvents used to solubilize polysaccharide, and experimental setup and processing parameters need to be taken into account collectively. The polymer structures and properties are important for electrospinning. For instance, molecular weight, through chain entanglements, dictates if the polymer solution can form fibers as it is being ejected from the syringe. However, too high of a molecular weight will make the solution highly viscous and unable to travel from the syringe nozzle to the collecting mandrel. The polysaccharide used for electrospinning can dictate the selection of a solvent (e.g., chloroform, hexafluoroisopropanol, trifluoroacetic acid, etc.). The glass-transition temperature is also important to consider as it could determine if the temperature within the electrospinning setup will affect the crystallinity and strength of the dry fibers that deposit on the collecting mandrel. Solution properties such as viscosity is controlled by the concentration and molecular weight of polysaccharide loaded into the solvent. To successfully electrospin a polymer, its concentration should be at least 2–2.5 times above the entanglement concentration (c_e_) so a continuous polymer fiber can be formed [60,61]. The surface tension and electrical conductivity play a role as to how charge within polysaccharides can induce stretching or beading effects within the fiber [62,63]. The electrospinning setup and its environmental conditions can influence fiber diameter, deposition, and alignment that have downstream effects on mechanical properties such as tensile strength and absorbency for removing wound exudate, necessary for a compliant wound dressing. The properties and parameters selected and how they mechanistically affect electrospinning are covered in more extensive reviews by Greiner and Reneker [64,65].

Polysaccharide biopolymers have become widely popular as electrospun materials because of their natural abundance, biodegradability, biocompatibility and antimicrobial properties. Polysaccharides that contain extracellular matrix derived glycosaminoglycans can be electrospun into non-woven matrices that mimic tissues being replaced during wound healing. Among the polysaccharides that have been electrospun (alginate, chitosan, dextran, cellulose, hyaluronic acid, starch, and heparin) as regenerative materials, this portion of the review will focus on chitosan with a comparison of its properties to other electrospun polysaccharides established in the literature. Chitosan nanofibers are suitable wound dressing materials because they offer inherent anti-microbial properties in addition to having biocompatible, biodegradable and hemostatic properties. Chitosan is a substrate for cell attachment due to their polymer structure exhibiting similarities to glycosaminoglycans (GAGs) that are a major component of the extracellular matrix [66]. Thus, the extent of application ranges from surgical sutures, artificial skin, and controlled drug delivery devices. Additionally, chitosan is derived from chitin, the second most abundant biopolymer on earth [67] thus, their availability can be leveraged to produce a low cost and effective wound dressing material. Unfortunately, chitosan can be difficult to electrospin because of its highly charged nature from deacetylation of its *N*-acetyl-d-glucosamine group that induces aggregation, making it difficult to solubilize in solvents [67]. The solubility of chitosan is controlled by molecular weight, solvent pH, acetyl distribution and acid used. Increasing molecular weight of chitosan increases the tendency of the electrospinning solution to become too viscous due to aggregation. Also, solvent pH of the electrospinning solution can be adjusted by introducing hydrochloric acid [68] and acetic acid [69]. Ideally, the pH of the electrospinning solution is below 6 but is affected by the degree of deacetylation (>60%) that causes solution insolubility [67]. 

There are a handful of solvents that dissolve chitosan into a solution that is successfully electrospun. Acetic acid (90 wt %), trifluoroacetic acid (TFA) and TFA/dichloromethane (DCM) are solvent systems typically used to electrospin chitosan [70]. A 1 wt % chitosan-silver nanoparticle composite in acetic acid was fabricated by Lee et al. dissolving the composite in a 7:3 TFA/DCM mixture at 5 wt % for electrospinning [71]. However, chitosan fibers lack stability in aqueous solutions and have limited electrospinning conditions used to successfully form fibers. Therefore, additional polymers are introduced to improve spinnability such as PEO, PVA, collagen and silk [5,7,70,72]. The most prominent example of successfully electrospinning chitosan without additives was done by Geng et al. who electrospun bead free fibers from a solution of 7 wt % chitosan (M.W. = 106,000 Da) in 90 wt % acetic acid [73]. Nonetheless, there is a major challenge developing chitosan electrospinning solutions that have a combination of low viscosity and high chain entanglement after dissolving. The Mark-Houwink equation, Equation (1) predicts molecular weight based on the intrinsic viscosity of the chitosan measured by a viscometer or rheometer [74]. 

The equation is expressed as follows:

(1) *[η]* = *KM^a^*

where intrinsic viscosity, *[η]* is related to the molecular weight (*M*) of the polymer being dissolved while *K* and a are constants that correspond to the intrinsic viscosity of the particular solvent system being used. Klossner et al. have established parametric constraints that create a rheology window for fiber formation from chitosan-polyethylene oxide blends based on acetic acid concentration, polymer concentration, and polymer molecular weight [75]. Further investigation probing the chitosan’s ionic properties, molecular weight, the degree of acetylation and acid solvent selection is necessary to find the optimal viscosity window to electrospin pure chitosan.

Other polysaccharides such as alginate and hyaluronic acid have been electrospun as wound dressing materials because of their biocompatibility. Alginate, like chitosan, is ionically charged but is a linear copolymer whose proportion of M-block and G-block content influence physical properties such as tensile strength and fluid absorption capacity [49]. Shalumon et al. created Alginate/Polyvinyl alcohol (PVA) blended nanofibers with zinc oxide as an anti-bacterial wound dressing [76]. Electrospun fibers from a mixture of alginate with two 37 kDa and 196 kDa molecular weights blended with polyethylene oxide (PEO) at ratios up to 80:20 in deionized water and surfactant [77]. However, their potential in electrospinning has not been fully realized because of existing challenges to fabricate uniform, continuous fibers. This is due to low chain entanglement created by negative charge repulsions and length of polymer chains within the alginate network [66,78,79]. As a result, groups introduced other polymers to assist in electrospinning such as PEO, PVA and glycerol to neutralize the electrostatic repulsions that promote greater fiber entanglement [66]. Hyaluronic acid has been reported to be successfully electrospun in dimethylformamide and water [80]. However, there has been limited success electrospinning HA alone due to its high charge density and surface tension. As a result, blended polymers are needed for it to be consistently electrospun successfully. Gelatin, PEO, and zein has been blended with HA to form fibers. Electrospun polysaccharide fibers are an effective platform that helps answer preclinical questions about extracellular matrix response to natural wound dressing models after implementation post-injury. Nevertheless, the scalability of electrospinning will need to be further refined. However, a new approach fabricating nanoscale fibers has been reported by Raoufi et al. to process hyaluronic acid feedstock solutions from a syringe extruded through nanoporous alumina membrane templates into uniform nanoscale fibers [81]. This technique may open up a new direction in the fabrication of polysaccharides wound dressing materials to such fine resolutions that can selectively affect critical processes during wound healing such as extracellular matrix activity and fluid absorption.

## 4. Biocompatibility Assessment of Polysaccharides as Wound Dressing Materials

### 4.1. Cytocompatibility Assessment

The biocompatibility of materials is the most important factor for wound dressing application as wounds can be potentially exposed to cytotoxic environments that would exacerbate the healing process. Consequently, it is important to ensure the wound dressing material itself is not inherently toxic so in vitro and in vivo assessment must be utilized to properly screen the materials selection process for cytocompatibility. Fortunately, for polysaccharides they are biocompatible because their origin in the extracellular matrix that plays a significant role during the wound healing process. Additionally, they are biodegradable and do not elicit an inflammatory immune response. Hyaluronic acid and chitosan are polysaccharides have primarily been explored as wound dressing materials. The cytocompatibility of these two polysaccharides among others will be reported from selected studies for this section.

Hyaluronic acid is a naturally occurring linear polysaccharide with repeating units of d-glucuronic acid and *N*-acetyl-d-glucosamine disaccharide [82]. It is a major component of extracellular matrix and is found in skin, cartilage, bone, and many other tissues [83]. Hyaluronic acid has been used commercially for wound dressings products under trade names Hyalomatrix, Hyalofill, Hyalogran, etc. using the ester based HYAFF-11 material developed by Anika Therapeutics. The commercialized dressings are esterification based hyaluronan materials offering cytocompatibility with fibroblast, keratinocyte, macrophage and complement proteins involved in inflammation. In animal models, it was discovered that the tissue response to HYAFF-11 implantation was mild with the presence of macrophages at the wound site 3–12 months post-implantation, indicating long-term biocompatibility [84]. HYAFF-11, in its native form, is a raw polymer that can be processed into tunable wound dressing material platforms such as hydrogels and foams. From the literature, it was reported hyaluronic acid hydrogels stimulate proliferation of fibroblasts that are responsible for collagen deposition and organization as fiber bundles [85]. Ji et al. reconstituted hyaluronic acid derivatives into electrospun fibers to serve as an ECM-mimicking substrate favorable for NIH3T3 fibroblast cell attachment and spreading, ideal for tissue regeneration [86].

Chitosan is an attractive wound dressing material because it is biocompatible, non-toxic, absorptive, antimicrobial, biodegradable, hemostatic, and can be a substrate for cell attachment [67,87]. As a wound dressing material, chitosan possesses bioadhesive properties on mucin substrates because of its positive charge at physiological pH [88]. Chitosan has also been shown to promote wound healing [89] and exhibit bacteriostatic effects by using its positive charge to bind to the bacteria’s cytoplasmic membrane [90,91,92]. During the remodeling stage of wound healing, chitosan accelerates healing and promotes smooth scarring at the injury site due to enhanced vascularization. Also, they possess a high supply of chitooligomers that incorporate collagen fibrils at the extracellular matrix [93,94]. Important wound healing mediators such as fibroblast growth factor (FGF-2) has been successfully integrated into chitosan hydrogels that better promote signaling mechanisms during the proliferation stage of wound healing [95]. Park et al. developed bFGF-loaded chitosan hydrogels to accelerate wound repair in chronic ulcers [96]. A UV-crosslinkable chitosan hydrogel system was fabricated by Ishihara et al. that occluded bleeding from the wound site while encouraging tissue granulation and epithelialization in rat models [95]. Good cytocompatibility and attachment of hepatocyte cells from liver tissues were demonstrated using electrospun nanoscale chitosan fibers, affording potential uses as a biomimetic ECM substrate for the liver [97].

Additional polysaccharides have been investigated for their biocompatibility as electrospun or hydrogel scaffolds that can be applied as a wound dressing platform. Vashisth et al. have reported the fabrication of amoxicillin impregnated electrospun gellan/polyvinyl alcohol composites as potential transdermal substitutes which revealed human keratinocyte cell adhesion and viability in vitro while encouraging skin re-epithelialization for in vivo animal models [98]. Dextran hydrogels are used as wound dressing materials they have exhibited angiogenesis and complete skin healing in animal burn wound models [99]. Sun et al. created excisions to full-thickness wounds before dextran hydrogel scaffolds were implanted with a secondary dressing layer for up to 21 days [99]. The study showed complete dermal regeneration after implantation of dextran hydrogels compared to non-treated and treated control groups. Our group has developed lyophilized arabinoxylan hydrogels that exhibited viabilities above 96% on fibroblasts cultured in vitro [44]. The results concluded that fibroblasts that play a prominent role in the proliferative and remodeling stages of wound healing maintain their cytocompatibility after exposure to arabinoxylan.

### 4.2. Bioactive Molecule Incorporation and Release

Advanced wound dressings developed more recently incorporate bioactive molecules to enhance patient comfort and to help accelerate the wound healing process. Antimicrobial dressings are one type of bioactive molecule important during the wound healing process that inhibits potential bacterial infections caused by acute tissue injury, post-operative surgery or from more chronic, pathological states such as diabetes [2]. Many anti-microbial dressings are impregnated with silver, which broadly acts against infections caused by skin burns and wounds. Silver has been the traditional antimicrobial agent to treat bacterial colonies such as *Staphylococcus* aureus and *P. aeruginosa* [48]. Its mechanism of action involves the influx of silver ions to the bacterial cytoplasm, where they shut down enzyme activity and as a result, potassium ions leak out the cell [100]. The released ions cause the cytoplasm to burst and destroy the cell wall, leading to apoptosis [100]. Silver can only be applied locally but has been effective inhibiting bacterial growth and its resistance [101]. However, silver’s spectrum of use should be limited because of its cytotoxicity. Therefore, the inherent antimicrobial activity of polysaccharides such as chitosan with less silver may be utilized as an alternative, less cytotoxic wound dressing.

Polysaccharide materials are ideal for bioactive molecule incorporation because their biodegradability can be controlled in the body based on their polymer structural properties, tuning bioactive release. Also, they are inherently bioactive that may serve as ligands, binding to receptors on the fibroblast’s surface during wound healing to promote extracellular matrix production [16]. Chitosan polysaccharides have extensively been used for the release of bioactive molecules. In a study led by Kumar et al., zinc oxide particles, known for their antibacterial activity were introduced into chitosan hydrogel bandages that reduced expression of *E. coli* bacteria in vivo. As a result of bacterial suppression, these bandages in vivo exhibited 90% wound closure in rat models two weeks post-injury [102]. Additional polysaccharides such as alginate was processed into fibers and impregnated with silver that exhibited antimicrobial effects against MRSA pathogens [103]. To improve post-surgical wound healing, researchers fabricated hyaluronic acid hydrogels crosslinked in situ and conjugated with anti-inflammatory agents dexamethasone [104] and bupivacaine [105]. Results from these studies revealed sustained release of these bioactive drugs in animal models. 

Arabinoxylan has shown potential as a drug delivery system as a gel by exhibiting high protein release. The rate of release can be modulated by the initial amount of protein loaded into the gel [41]. Crosslinked arabinoxylan gels have high water absorption capacity that enables potential drug delivery applications using therapeutics such as albumin and ibuprofen [41,106]. Our previous work demonstrated arabinoxylan foams impregnated with silver can effectively serve as an in vitro antimicrobial wound dressing material [44]. Alginate-pectin aerogel particles with pectin core shells have encapsulated doxycycline antibiotic in wound dressings for sustained drug release against tissue degrading MMPs during chronic wound healing [107]. While current trends of integrating broad-spectrum antimicrobials into polysaccharide materials have been established and have been effective preventing chronic wounds, utilizing strategies to incorporate less toxic bioactive agents with greater selectivity is needed for more effective wound healing management. Extensive work incorporating bioactive molecules such as epidermal growth factor, vitamin, and arginine in hyaluronic acid sponges proved to promote inflammation and wound closure in animal models [108,109]. Polysaccharides containing naturally derived agents may serve as a viable bioactive alternative for wound healing. Naseri et al. electrospun a 1:1 blend mixture of chitosan:PEO with chitin nanocrystals as a potential wound dressing material [110]. A summary of these naturally derived molecules is beyond the scope of this paper but covered by Laurienzo et al. in another review [111].

## 5. Conclusions

In an increasing health-cognizant society, there is a greater demand for naturally derived materials for medical treatment. Wound dressing development is advancing at a rapid pace because of integrating naturally derived materials such as polysaccharides. Polysaccharides have been primarily used in food, textile or cosmetic products, but their potential utility as wound dressings is vast because of their abundance and non-toxicity. These class of polymers is excellent candidates for wound dressing development because they exhibit a structural diversity of molecular weights and structures that influence their overall material properties. Many of the polysaccharides described in this review are well established in vitro and in vivo as hydrogel and electrospun wound dressing platforms. There has been work using more selective fabrication techniques such as designing enzymes to functionalize polysaccharides and influence their anti-microbial, fluid retention and gel strength properties [112]. Cellulose polysaccharides have been widely researched and have potential clinical uses; however, this review does not cover these class of materials that is extensively covered in a review by Czaja et al. [113]. 

An exciting time for polysaccharides wound dressing materials is ahead through the expansion of manufacturing processes such as 3D printing, with the potential to create patient-specific wound dressings with design freedom using computerized models. 3D printing of polysaccharides is realized by a process called bioprinting that uses selective deposition of a gelatinous ink in three-dimensional space to create controlled geometric structures. Pescosolido et al. have incorporated hyaluronic acid and dextran into crosslinked hydrogels that were reprocessed into three-dimensional scaffolds using bioprinting [114]. A very recent review on bioprinting alginate, covering its state of the art and challenges was published by Axpe et al. [115]. Polysaccharides are naturally derived materials intended to improve bioactivity and tailored performance of commercialized wound dressings. The authors hope this review will spur further investigation and development of polysaccharides as a natural source of wound dressings materials.
